# Breast Cancer Risk After Benign Breast Disease Among Racially Diverse Women: Systematic Review and Meta-Analysis

**DOI:** 10.21203/rs.3.rs-10475905/v1

**Published:** 2026-08-04

**Authors:** Alzina Koric, Nusrat Preema, Akila Anandarajah, Shivani Patel, Angela Hardi, Graham A. Colditz

**Affiliations:** Washington University in St. Louis; Washington University in St. Louis; Vanderbilt University; St. Joseph Medical Center; Washington University in St. Louis; Washington University in St. Louis

**Keywords:** Benign breast disease, breast cancer, meta-analysis, race, ethnicity, worldwide

## Abstract

A meta-analysis of the existing literature was conducted to quantify breast cancer risk according to histologic subtype of benign breast disease (BBD) among women of diverse racial and ethnic backgrounds worldwide. PubMed (Medline), EMBASE (Emtree), and other relevant biomedical databases were searched through February 2026 to identify studies reporting the development of subsequent breast cancer in women diagnosed with either nonproliferative disease (NP), proliferative disease without atypia (PDWA), or atypical hyperplasia (AH). Seven studies met the eligibility criteria. Women of Asian ancestry with PDWA or AH had a substantially higher risk of breast cancer than those with NP. Among Black women, breast cancer risk associated with BBD subtypes was comparable to historically reported risks among White women, whereas associations among Hispanic women were less clear due to greater data limitations. The variable BBD-associated breast cancer risk across racial groups warrants confirmation and underscores the need for further investigation of the mechanisms underlying these differences.

## INTRODUCTION

Breast cancer remains a leading malignancy among women worldwide, with an estimated 2.3 million new cases and 670,000 deaths in 2022.^1^ By 2050, incident cases are projected to increase by 38% and deaths by 68%, driven in part by the aging population and lifestyle changes.^2^ Racial and ethnic disparities in breast cancer incidence and mortality persist, with highest incidence among Black, Asian/Pacific Islander, Hispanic, and American Indian/Alaskan Native women, second to those observed among White women.^3^ Despite having similar incidence rates, Black women have the highest breast cancer mortality, with rates 42% higher than those among White women.^3^ Benign breast disease (BBD) represents a critical window for breast cancer risk stratification and preventive intervention, particularly in the presence of epithelial proliferation or atypia. Diagnosed by biopsy and classified by histologic risk, atypical hyperplasia (AH) is identified in up to 10% of women and confers a 4–5-fold increased risk of future invasive disease.^4–9^ Proliferative disease without atypia (PDWA), which accounts for approximately 40% of benign lesions, is associated with a moderate increase in risk of breast cancer (2-fold), while non-proliferative disease (NP) carries a slightly elevated risk compared with women who have never undergone biopsy.^4,6–9^ However, existing evidence is derived from predominantly White women and does not fully align with the limited, small-sample studies conducted in women of diverse racial and ethnic backgrounds.

AH-related breast cancer risks in individual studies range from 3.3–3.5-fold in Black women,^10,11^ 3.8–4.5 in Hispanic women,^12,13^ and from 5–33-fold in Asian women.^11,14,15^ PDWA-related risks are similarly variable, with a 5–7-fold increase in breast cancer observed in Asian women^11,15^ and about 1.5-fold increase in Hispanic women;^11,12^ although, estimates derive from a limited number of small-sample studies. In contrast, the existing US studies among Black women, reported PDWA risk as 1.01–1.16-fold,^10,16^ but the estimates were not statistically significant. In view of the limited data available from diverse large-scale, population-based studies, aggregation of data across multiple existing smaller studies can enhance statistical power and produce robustness of study findings, to elucidate the association between benign breast histology and subsequent breast cancer risk. Thus, the objective of this meta-analysis was to quantify the risk of breast cancer by histologic subtypes of benign breast lesions (i.e., PDWA, AH) among women of diverse racial and ethnic backgrounds worldwide.

## METHODS

### Study Eligibility

The eligibility criteria of studies included in this meta-analysis were: a) histologically confirmed benign breast lesions among women of diverse ethnicity or race backgrounds, b) outcomes of invasive or in-situ breast cancers reported by the key histological subtypes (i.e., NP, PDWA, AH) and recorded within 6 months, if available, and c) observational cohort or case-control designs. Studies without outcome count by histologic subtype were excluded. Published abstracts with sufficient histology information were included in the analysis. Restrictions on publication date or language were not applied.

This study was based on previously published literature and aggregated summary data. Institutional Review Board (IRB) review approval from the Washington University School of Medicine was not required for this meta-analysis and systematic review.

### Study Selection and Research Strategy

This meta-analysis was prepared in accordance with the *Preferred Reporting Items for Systematic Review and Meta*-*Analysis* (PRISMA 2020) guidelines.^17^ The published literature was searched using terms developed by a medical librarian (AH), combining controlled vocabulary and keywords in PubMed (Medline) and EMBASE (Emtree) as well as Ovid-Medline, EBSCO, Scopus, Web of Science, and the Cochrane Library on February 24, 2026 (date of the last search). Complete search strategy is provided in the supplemental materials available online. The selection of a systematic literature of eligible studies included in this meta-analysis is presented in the PRISMA flow diagram available online (Supplementary Fig. 1). Independent screening of the identified studies was performed by three authors and discrepancies were resolved by discussion between the lead and corresponding author (AK, GC). The initial review of eligible studies was based on the relevance of titles and abstracts, followed by a full text review of the remaining studies using the inclusion criteria. EndNote and Covidence software^18^ were used for optimal management and screening of eligible studies. Reference list of eligible articles were screened for additional relevant literature. For studies of the same cohort with overlapping diagnosis year, the study with the largest sample and available BBD subtype information was used in the analysis.

### Outcome Measures

The main outcome is subsequent risk of breast cancer (invasive and in-situ) following a proliferative benign disease (PDWA and AH, exposures) diagnosis compared with women diagnosed with nonproliferative benign lesions (NP, as the reference) overall and stratified by race.

### Information Extraction and Data Items

Available information from each eligible study was extracted by two readers on study and participant demographics including: lead author’s name, publication date (year), study design, age at biopsy (range and mean/median), study location, total women at risk in each BBD histologically confirmed subtype, number of cases (i.e., breast cancer) in each BBD subtype, and follow-up time (mean/median) for the overall cohort of women diagnosed with BBD. For studies using women without BBD as the comparison, only information on BBD subtype in the populations of interest (i.e., Black, Hispanic, Asian) was extracted. For each study, the number of women by BBD subtype and the corresponding number of breast cancer cases for each was extracted. The risk estimates were calculated from the raw counts of women with and without subsequent breast cancer to allow for standardized comparisons across studies using the NP group as the reference, rather than relying on standardizing the varied risk measures reported in the original studies, some of which also used women without BBD as their comparison group. However, original risk estimates from each study were extracted and assessed on a log scale and summarized for transparency and completeness of reporting and preserving original study adjusted effects. Relevant information of broader benign lesions reported in studies were grouped by standard BBD subtype categorizations (i.e., NP, PDWA, AH).^19,20^ Data on unidentified BBD subtypes were extracted for assessment as a distinct unknown category of benign lesions when compared with NP. The extracted individual study data were double-checked by the lead author for accuracy.

### Analytical Approach

To synthesize the information from individual studies, a random-effect model was used to estimate pooled odds ratios (ORs) with 95% confidence intervals (95%CI) for the association between the histologic subtypes of benign breast lesions and subsequent breast cancer risk (binary outcome). The risk of breast cancer for PDWA and AH compared with NP subtype, overall and stratified by race, were presented in a forest plot. To preserve study level adjusted effects for confounding, we also pooled the original study-reported estimates (Supplementary Table 1) and reported these associations on the log scale as a sensitivity analysis. Further sensitivity analyses were conducted excluding studies that did not clearly report the onset of cases, typically defined as within 6 months following benign breast biopsy. Study-level age was estimated and when only the median age was reported, the mean was estimated using the Hozo method.^21^ For studies that reported neither mean nor median, the midpoint of the reported age range was used to approximate study-level mean age at biopsy.

The between-study heterogeneity across eligible studies was assessed with the Cochrane’s Q and *I*^2^ statistics.^22^ The Q statistics was used to test between-study heterogeneity, at p < 0.10 statistical significance. The *I*^2^ test describes the proportion of total variation that is due to heterogeneity between studies, with 30–60% indicating moderate acceptable heterogeneity for the pooled OR assessment. As per the current practice,^22,23^ the random-effects model was used if there was heterogeneity across studies or if the Q > K–1(df) and I^2^ >30% given the true effect size may vary across studies, rather than assuming a single, common effect size existed for all studies suitable for the fixed-effects model. Thus, the effect sizes in individual studies were assumed to be a random sample from a distribution of true effects, allowing for heterogeneity (variation) among studies. Heterogeneity was further explored using leave-one-out analyses to assess the impact of individual studies on the I^2^ estimates. Publication bias was assessed with Egger’s test of asymmetry determined by the t-test at p < 0.05 statistical significance as well as graphically with a funnel plot for PDWA and AH subtypes of benign lesions individually, with a symmetrical plot indicating no publication bias. Methodological quality of eligible studies was evaluated with the Newcastle-Ottawa Quality Assessment Scale (NOS).^24^ All analysis were performed with STATA software version 17.0 (Stata Corporation, College Station, TX, USA).

## RESULTS

A total of 1,013 results were retrieved from the initial database literature search, and 538 duplicate citations were removed. The remaining 475 citations were screened, resulting in 10 studies that initially appeared to meet the inclusion criteria. However, two studies that did not report total number of cases for race by BBD subtypes were excluded,^25,26^ and another study was excluded because it assessed immediate pathologic upgrades (primarily in situ cases) on excision rather than tracking women with BBD for subsequent breast cancer outcomes.^27^ Thus, a total of seven eligible observational cohort studies were included in this meta-analysis of women diagnosed with histologically confirmed BBD worldwide.^10–16^ However, in our prior study that included Black and Asian women,^11^ each subgroup was treated as a separate study in the meta-analysis.

The included studies were of good methodological quality (Supplementary Table 2), with consistent strengths in cohort selection and outcome ascertainment representative of the women with benign lesions identified in medical records with 6.4 to 12.8 mean or median years following BBD. One study included a contemporary cohort of women biopsied in the recent years,^11^ three studies included women biopsied before 2015,^12,13,16^ and the rest included women who were biopsied before 2000.^10,14,15^ The total sample size across studies ranged from 299 to 9,223 among Black, Hispanic, and Asian women diagnosed with BBD worldwide between 1972 and 2023. Total cases of women diagnosed with breast cancer ranged from 5 to 247. The pooled median age at biopsy was 49.25 years (range 18–90, mean age = 49.41, SD ± 4.64 and 52.12 ± 3.80 (median = 51.75) based on midpoint age range) based on all 7 studies and age summary distribution from each study was reported in Table 1.

Of the eligible studies summarized in Table 2, two studies, one in the US and the other in Spain, evaluated breast cancer risk in Hispanic women according to BBD histology.^12,13^ There were 3 studies in the US that evaluated breast cancer risk in Black women according to BBD histology.^10,11,16^ The Cote et al. 2012 study was included for the analysis of AH subtype only, because this and the Patil et al. 2024 US study among Black women overlapped for the assessment of PDWA subtype. Finally, 2 studies from Japan and the previously described US study evaluated breast cancer risk in Asian women.^11,14,15^

**Figure 1:**
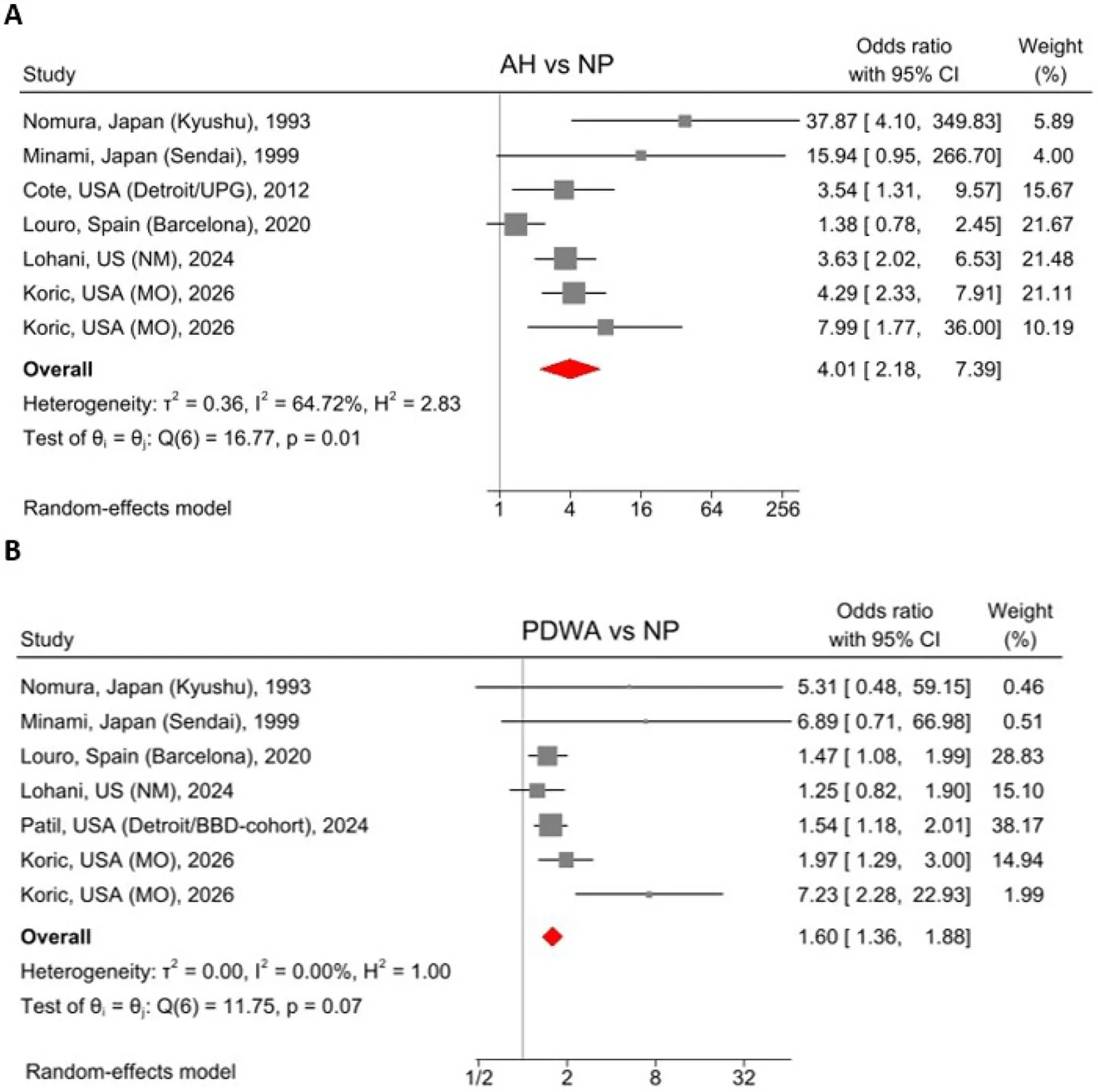
Forest plot for meta-analysis of subsequent breast cancer risk following BBD for women diagnosed with (**A**) atypical hyperplasia (AH) and (**B)** proliferative disease without atypia (PDWA) diagnosed between 1972–2023

In the primary analysis, overall pooled ORs were recalculated from raw counts for each study, yielding a 4-fold AH-associated breast cancer risk compared with NP (OR = 4.01, 95%CI 2.18, 7.39, I^2^ = 64.72%, n = 7, Fig. 1). In contrast, exclusion of 2 studies without clear separation of cases within 6-months of follow-up gave an OR = 4.15, 95%CI, 2.86, 6.03, I^2^ = 0.00%, n = 5 (Supplementary Fig. 2). Overall PDWA was associated with increased breast cancer risk compared with NP (OR = 1.60, 95%CI 1.36, 1.88, I^2^ = 0.00%, n = 7, Fig. 1). Of note, after exclusion of 2 studies without clear separation of cases diagnosed within 6-months following benign biopsy the OR was OR = 1.98, 95%CI 1.21, 3.25, I^2^ = 74.90%, n = 5 (Supplementary Fig. 3). Overall, heterogeneity in the studies evaluating PDWA was low (I^2^ = 0.00%, n = 7, p = 0.75), whereas studies evaluating AH showed moderate acceptable heterogeneity (I^2^ = 64.73%, df = 7, p = 0.01, Fig. 1). With preserved adjustments of the original risk estimates in the sensitivity analysis, the AH-associated risk estimates were similar to those observed in the primary analysis, with and without inclusion of the 2 studies with unclear 6-month timeframe of cases (Supplementary Figs. 4 & 5). Whereas, PDWA-associated risk estimates were considerably higher in the sensitivity analysis with substantial heterogeneity (Supplementary Fig. 5).

**Figure 2:**
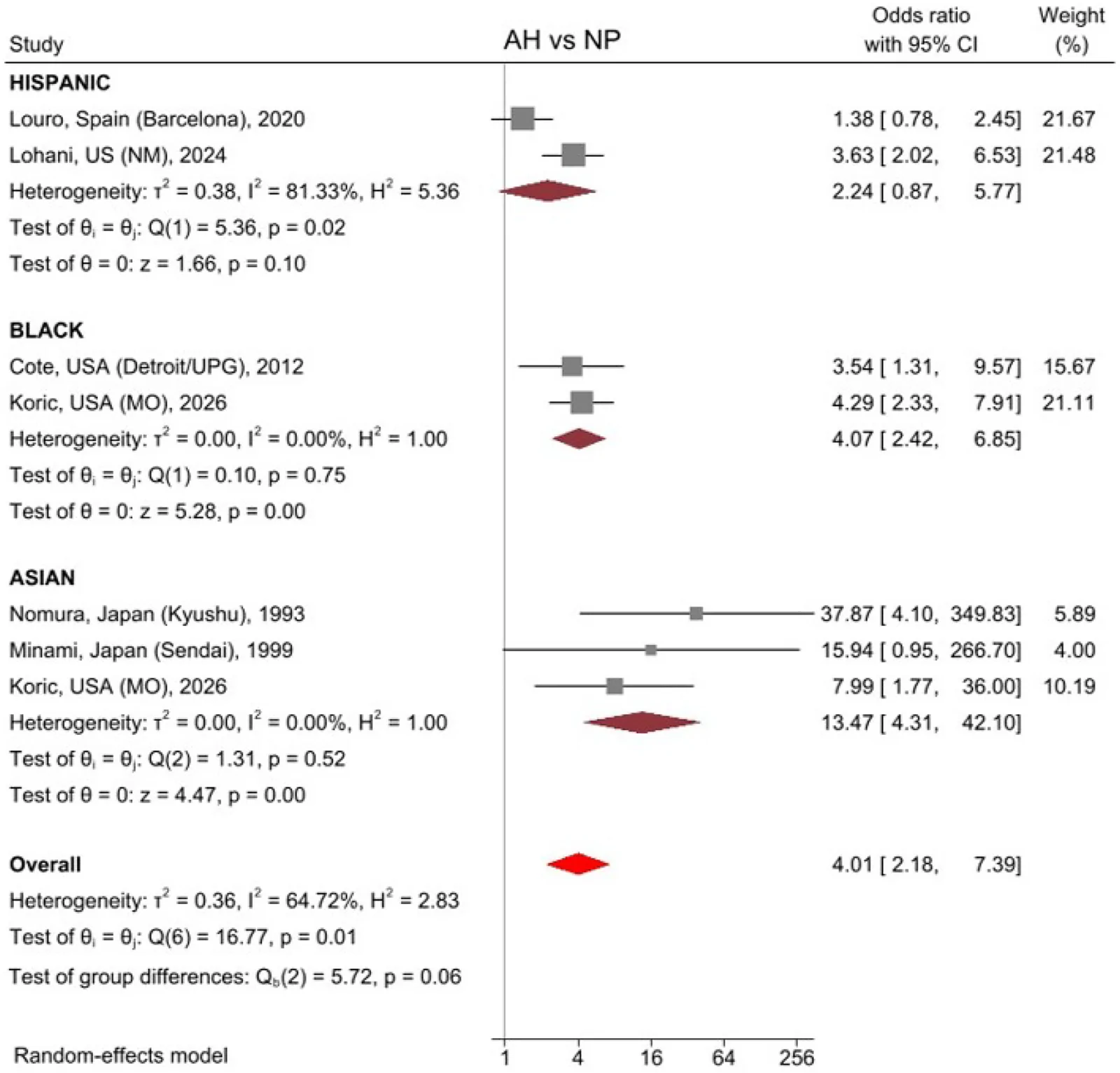
Forest plot for meta-analysis of subsequent breast cancer risk following BBD for women diagnosed with atypical hyperplasia (AH) compared with nonproliferative (NP) lesions between 1972–2023, presented by race and ethnicity

**Figure 3:**
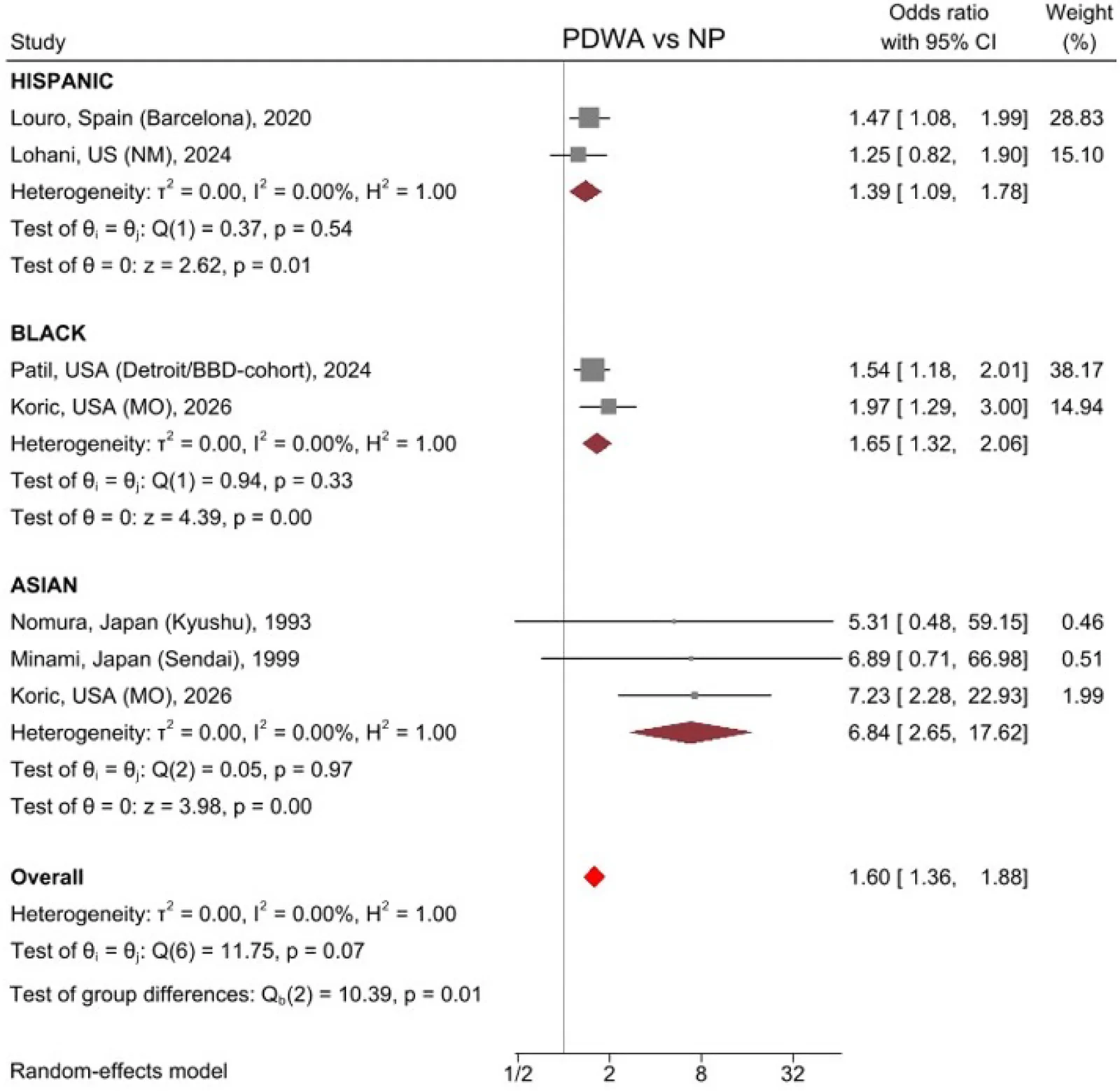
Forest plot for meta-analysis of subsequent breast cancer risk following BBD for women diagnosed with proliferative disease without atypia (PDWA) compared with nonproliferative (NP) lesions between 1972–2023, presented by race and ethnicity

Within race groups, an elevated pooled OR of AH was observed in Asian (OR = 13.47, 4.31, 42.10, heterogeneity: I^2^ = 0.0%, n = 3) and Black (OR = 4.07, 2.42, 6.85: I^2^ = 0.0%, n = 2), but not Hispanic women (OR = 2.24, 95%CI 0.87, 5.77: I^2^ = 81.33%, df = 2, Fig. 2). PDWA-race associated breast cancer risks were observed in Asian (OR = 6.84, 95%CI 2.65, 17.62, I^2^ = 0.00%, n = 3), Black (OR = 1.65, 95%CI 1.32, 2.06, I^2^ = 0.00%, n = 2), and Hispanic women (OR = 1.39, 95%CI 1.09, 1.78, I^2^ = 0.00%, n = 2, Fig. 3). In the sensitivity analysis with preserved original adjusted estimates, similar breast cancer risk estimates to the primary analysis were observed, except PDWA-associated risks were not significant for Black women. PDWA-associated risks had substantial heterogeneity, reflecting differences in study populations and adjustment for breast cancer risk factors.

The removal of one study at a time in the sensitivity analysis did not substantially alter the originally observed OR estimates (Supplementary Table 3). Egger’s test of asymmetrical evidence suggested evidence of small-study effects (β = 1.81, p = 0.010), but the test is underpowered with only seven studies. Publication bias was observed visually with the funnel plot indicating that smaller studies reported larger effect sizes, consistent with the regression findings (Supplementary Fig. 6); however, a minimum of 10 studies is considered appropriate for the funnel plot analysis.

## DISCUSSION

In this meta-analysis of observational cohort studies, the observed association between BBD histology and subsequent breast cancer risk may not be uniform across racial groups. These findings should be interpreted as preliminary signals of potential heterogeneity in risk trajectories that warrant further consideration. While risk estimates among Black women with AH and PDWA were broadly consistent with those historically derived from predominantly White populations, the substantially elevated risk estimates observed among Asian women, regardless of histologic subtypes or world region, suggest the existing patterns in risk may be systematically underestimated in this population. The observed findings among Hispanic women were less consistent, reflecting sparse data and methodological variability in the included studies of Hispanic women with BBD in this meta-analysis. The relatively younger age of women included in meta-analysis further suggests that BBD diagnosis may represent a critical window for early risk stratification and targeted prevention, particularly in the context of rising breast cancer incidence among younger women.

The notably elevated breast cancer risk estimates observed among Asian women following both PDWA and AH are particularly relevant in light of recent epidemiologic trends showing rising breast cancer incidence in Asian women.^28^ Although the relatively small number of contributing studies and events in this meta-analysis raises the possibility of inflated effect estimates, the findings may also reflect underlying biological or diagnostic factors driving breast cancer risk following BBD in Asian women. High prevalence of dense breast tissue has been associated with to the elevated risk of breast cancer among Asian women.^29^ However, Burton et al. 2017 and colleagues^30^ observed that age- and menopause-related changes in breast density are broadly similar across populations, suggesting that differences in breast cancer risk following BBD are unlikely to be explained solely by divergent density trajectories. Instead, racial differences in breast cancer risk following BBD likely reflect variation in baseline breast density, screening and detection patterns, and other biological or environmental factors. Consistent with this interpretation, the Burton study identified ethnic differences in breast size, density levels, BMI, and reproductive characteristics, despite similar age-related declines in breast density. More broadly, emerging evidence suggests that breast cancer progression pathways vary across populations. For example, a recent molecular study found that mechanisms of progression following ductal carcinoma in situ differ between Black and White women.^31^ Given limited comparable evidence for BBD for Asian, Hispanic, and other racial and ethnic groups, there is a need to investigate pathway-specific mechanisms across diverse populations of women with BBD to determine whether differences in subsequent breast cancer risk reflect distinct biologic processes that could inform risk stratification and prevention strategies.

In contrast, the risk estimates observed among Black women were consistent with prior literature, suggesting that existing clinical management strategies derived from predominantly White populations may be broadly applicable to this group. Nevertheless, our pooled risk estimates identified a modest (1.5-fold) but statistically significant increase in breast cancer risk among Black women with PDWA, in contrast to prior individual studies reporting null associations (RR = 1.01, 95%CI 0.71–1.45 from Patil et al. 2024 and RR = 1.16, 95%CI 0.64, 2.12 from Cote et al. 2012).^10,16^ These finding underscores the value of data aggregation for Black women in improving statistical power and suggests that PDWA should not be considered a negligible risk factor in this population. Similarly, for Hispanic women, although individual studies have primarily reported an increased breast cancer risk following BBD relative to women without BBD,^12,13^ our pooled estimates suggest that PDWA confers increased breast cancer risk when compared with NP subtype. Taken together, these results indicate that risk of breast cancer following BBD may vary across racial and ethnic groups, underscoring the need for population-specific risk assessment and further investigation of the factors underlying these differences.

From a clinical perspective, the heterogeneity in subsequent breast cancer risk observed among BBD highlights both opportunities and limitations in current approaches to breast cancer risk stratification and management. Although over one million women undergo breast biopsy annually in the US, > 75% diagnosis are benign pathology,^32^ and the treatment of BBD lacks evidence-based recommendations to guide surgical, screening, or pharmacologic interventions for breast cancer risk reduction. For AH, the American Society of Clinical Oncology recommends risk-reduction endocrine therapy for selected high risk patients,^33^ as does NCCN.^34^ The American Society of Breast Surgeons Choosing Wisely recommendations emphasize avoiding unnecessary interventions (e.g., no routine excision of small fibroadenomas), with care guided by symptoms and imaging rather than routine surgery.^35^ Still, high-level prospective evidence and standardized management lead to variability in practice, especially how to individualize prevention strategies.

This study has several limitations. First, the small number of cases within race-specific BBD subtype categories limited statistical power resulting in wide confidence intervals particularly for the assessment of AH. By pooling data from the available studies and harmonizing the definition of the reference group, we provide a more precise estimation of the association between BBD subtype and the development of breast cancer. Second, while all studies included in this meta-analysis are of cohort design, methodological differences remain across studies that may have contributed to residual heterogeneity in the pooled estimates. Third, the inability to disaggregate broad racial categories, particularly among Asian populations, limits the interpretability of findings for these heterogeneous groups. Fourth, potential publication bias and small-study effects may have contributed to the larger effect sizes observed in smaller studies, thus these limitations should be considered when interpreting the results.

Despite these limitations, to our knowledge this study provides a novel synthesis of racially-associated breast cancer risk following BBD histology. The consistency of findings in sensitivity analyses supports the overall robustness of the primary results, while the use of a uniform reference group enhances comparability across studies. Additionally, the use of both raw count–based estimates and pooling study original adjusted estimates enhances the robustness and transparency of the observed findings. The observed high heterogeneity in the analyses of PDWA with the use of original adjusted study effects reflects differences in covariate adjustment across studies rather than analytical inconsistency. By contrast, AH-associated risk estimates across race groups were comparable to those in the primary analysis with minimal heterogeneity under the same approach. While, studies evaluating Asian women had minimal heterogeneity for PDWA and AH, although a statistically significant result may indicate a problem with heterogeneity, our non-significant results must not be taken as evidence of no heterogeneity. The observed results can be interpreted with an understanding of good methodological quality of all the studies in this meta-analysis. Because the inclusion of all BBD women selected from registries indicates the BBD cohort is drawn from the same source as the exposed cohort (e.g., PDWA, AH) and is truly representative of the average women in the respective cohorts across all the included studies. The effect of time remains a critical area for future research to determine whether breast cancer risks associated with BBD among women across diverse populations are comparable to those reported among White women, for whom studies have reported elevated risks over a decade after biopsy.^36–38^ This is important because the timing of cancer diagnosis can influence screening strategies, surveillance intensity, and discussions around preventive interventions. It is also relevant in the context of potential cohort effects, as evolving reproductive patterns and lifestyle risk factors^2^ may continue to drive increases in breast cancer incidence.

In conclusion, this meta-analysis suggests that breast cancer risk following BBD may differ across racial groups, the absolute greatest risk observed among Asian women with atypical hyperplasia, followed by Black women, and of unclear associations among Hispanic women. However, these results should be interpreted with caution given the current data limitations. Still, the younger age of women in these studies highlights BBD as a potential window for early, risk-informed intervention. As breast cancer incidence continues to rise among younger women, improving risk stratification across diverse populations will be essential for targeted prevention and reducing disparities in breast cancer outcomes.

## Supplementary Material

Supplementary Files

This is a list of supplementary files associated with this preprint. Click to download.

- bbdsrmasupplemental.docx

## Figures and Tables

**Table 1:** Characteristics of observational cohort studies worldwide with histologically confirmed BBD included in this meta-analysis, stratified by race and presented chronologically (1972–2023)

Author/Race	World region	Year published	Age range	F(t)^a^	BBD diagnosis	With breast cancer				Without breast cancer			
						NP	PDWA	AH	Total	NP	PDWA	AH	Total
**Hispanic**													
Lohani^b^	USA, NM	2024	20–89	12.8	1996–2007	73	33	15	121	2,087	755	15	2,857
Louro^f^	Spain, Barcelona	2020	50–69	7.8	1994–2015	177	57	13	247	7,053	1,548	375	8,976
**Black**													
Koric^c^	USA, MO	2026	18–87	7.0	2010–2023	61	36	14	111	2,056	616	110	2,782
Patil	USA, MI/MDCSS	2024	18–81	6.4	1997–2010	114	124	na	238	1,889	1,334	na	3,223
Cote^d^	USA, MI/MDCSS	2012	20–84	10.1	1997–2000	33	17	5	55	912	388	39	1,339
**Asian**													
Koric^c^	USA, MO	2026	18–76	4.9	2010–2023	5	8	3	16	219	49	15	299
Minami^e^	Japan, Sendai	1999	40–63	7.6	1978–1986	1	3	1	5	255	111	16	382
Nomura^f^	Japan, Kyushu	1993	30–70	8.0	1972–1987	1	2	4	7	284	107	30	421

Abbreviations: AH, atypical hyperplasia; BBD, benign breast disease; F(t), follow-up time; NP, nonproliferative disease; PDWA, proliferative disease without atypia; na, missing data;

a Mean or median follow-up time

b Unidentified BBD subtype, not included in the analysis (n = 603)

c Population of Black and Asian women from the same study cohort but separate estimates were generated for Black and Asian women, each subgroup was treated as a separate study in the meta-analysis.

d Included for the analysis of AH only since Cote and Patil overlap for the assessment of the PDWA subtype of benign lesions

e Invasive breast cancer diagnosis only at 12 months after BBD biopsy, not including ductal carcinoma in situ

f Unclear separation of cases within 6-months of follow-up following BBD biopsy

**Table 2:** Summary of age from observational worldwide BBD cohort studies included in this meta-analysis, stratified by race and presented chronologically (1972–2023)

Author/Race	World region (BBD diagnosis yr)	Sample	Median & mean (± SD) cases/non-cases	Age range
**Hispanic**				
Lohani^a^	USA, NM (1996–2007)	2978	46	20–89
Louro	Spain, Barcelona (1994–2015)	9223	na	50–69
**Black**				
Koric^b^	USA, MO (2010–2023)	2893	50 & 50 (13.5)	18–87
Patil	USA, MI/MDCSS (1997–2010)	3461	47/51	18–81
Cote	USA, MI/MDCSS (1997–2000)		48.6 (na)	20–84
**Asian**				
Koric^b^	USA, MO (2010–2023)	299	46 (11.9)	18–76
Minami	Japan, Sendai (1978–1986)	387	45.1 (na)	40–63
Nomura	Japan, Kyushu (1972–1987)	428	45.9 (0.5)/45.7(0.3)	30–70

Abbreviations: BBD, benign breast disease; na, missing data

a Unidentified BBD subtype, not included in the analysis (n = 603)

b Population of Black and Asian women from the same study cohort

## Data Availability

All data for this study are available in the paper and supplementary information. The data can also be provided in excel format upon request.
